# RhoA with Associated TRAb or FT3 in the Diagnosis and Prediction of Graves' Ophthalmopathy

**DOI:** 10.1155/2022/8323946

**Published:** 2022-07-29

**Authors:** Sidi Zhao, Shuangshuang Shi, Wanchen Yang, Hanqing Wang, Tianming Jian, Qing He, Yang Liu, Xiaoming Huang, Tong Wu

**Affiliations:** ^1^Tianjin Key Laboratory of Retinal Functions and Diseases, Tianjin Branch of National Clinical Research Center for Ocular Disease, Eye Institute and School of Optometry, Tianjin Medical University Eye Hospital, Tianjin 300384, China; ^2^Department of Ophthalmology, First Hospital of Qinhuangdao, Qinhuangdao, 066000 Hebei Province, China; ^3^Department of Endocrinology, Tianjin Medical University General Hospital, Tianjin 300041, China; ^4^Research and Development Department, Microsensor Labs, Chicago, IL 60602, USA; ^5^Sichuan Eye Hospital, AIER Eye Hospital Group, Chengdu, Sichuan Province, China

## Abstract

During Graves' disease (GD) treatment, Graves' ophthalmopathy (GO) is often ignored because only mild ocular symptoms are present in early GD. Therefore, we performed isobaric tags for relative and absolute quantification (iTRAQ) analysis and measured relevant endocrine hormones to identify predisposing factors of GO. Serum samples from 3 patients with mild GD and GO and 3 patients with GD but without GO were analyzed by iTRAQ. Based on their clinical data, 60 patients with GD were divided into the GO-free and GO groups. All patients were followed up for 7 months. Their eye conditions and changes in related biochemical indexes were recorded. The iTRAQ results showed that RhoA expression was upregulated and correlated significantly with the tight junction pathway and immunity. The changes in FT3 and RhoA from baseline to 7 months, the FT3 and RhoA baseline levels, and the TRAb titer levels in patients with GD significantly differed between the groups. ELISA and western blotting for RhoA, TRAb, and FT3 in the serum samples from GO patients showed significant upregulation, as well as elevated serum RhoA and TRAb levels in the mild stage of GO. At 7 months, the serum RhoA and FT3 levels were elevated. RhoA is a potential biomarker for mild GO. In GD patients, if an elevated serum RhoA level is accompanied by an elevated TRAb or FT3 level, GO is highly likely to occur, even when obvious ocular symptoms are absent.

## 1. Introduction

Graves' disease (GD) is an autoimmune thyroid disease and the main cause of hyperthyroidism in adults. Incidence rates are approximately 3% for females and 0.5% for males [1, 2]. Graves' ophthalmopathy (GO) is the most common extrathyroidal manifestation. Approximately 25%-50% of GD patients have ocular involvement [3, 4]. Most GO patients have hyperthyroidism, though a small number of cases may be hypo- or euthyroid [5–7]. Approximately 90% of GO patients display symptoms of eyelid retraction, which is an initial symptom and sign of early disease [8, 9]. Approximately 3% to 5% of GO patients will develop severe disease, which involves severe inflammation, eye pain, and vision-threatening corneal ulcers or compressive optic neuropathy (CON) [3, 4, 10, 11]. The appearance of GO is often ignored due to the presence of only mild ocular symptoms in the early stage.

Proteomics is a large-scale, high-throughput, and systematic study of the composition and function of all proteins in a certain type of cell, tissue, or body fluid. High-quality protein separation and identification technology combined with full utilization of bioinformatics reflects changes in the composition and expression level of proteins as a whole, which helps to identify those related to the formation and development of diseases and to explore the process of disease occurrence and development. Biological mechanisms and clinically related research as well as research and development of new drug targets, among others, have been widely employed [12–14]. In recent years, high-throughput mass spectrometry has been implemented and used in the study of screening serum proteins, revealing many molecules with biomarker values.

In the past few decades, the incidence of GO has shown an increasing trend. GO severely affects vision, leading to blindness and depression in most patients. Therefore, treatment early in the mild stage of the disease is likely to result in a better prognosis. The purpose of this study was to determine risk factors leading to the onset of GO, which is very important for its early diagnosis. We observed changes in levels of endocrine hormones in GD patients and discovered possible markers for early diagnosis of GO through proteomics, with a view toward early diagnosis of CON and prevention of visual impairment caused by it. We sought to provide new ideas for improving the diagnosis and treatment of GO and for evaluating prognosis.

## 2. Materials and Methods

### 2.1. Subjects

We collected clinical data and serum samples for GD patients admitted to the Tianjin Medical University General Hospital from June 2018 to October 2019. We randomly collected venous blood from 3 patients in the mild stage who were first diagnosed with GO complicated with GD (experimental group) and 3 diagnosed with GD without GO (control group) treated at the Tianjin Medical University Eye Hospital from January 2020 to October 2020. The present study was approved by the Tianjin Medical University Eye Hospital Foundation Institutional Review Board (REC No. 2020KY(L)-01) and followed the principles of the Declaration of Helsinki. Written informed consent was obtained from all study subjects. The study participants were not financially compensated.

Diagnostic criteria of GD were as follows [1, 2]: (1) clinical diagnosis of hyperthyroidism, (2) diffuse thyroid enlargement (confirmed by palpation and B-ultrasound), with some cases lacking goiter, (3) exophthalmos and other infiltrating eye signs, (4) anterior tibial mucin edema, and (5) TRAb, TSAb, and TPOAb positivity. Of the above criteria, items (1) and (2) were essential for diagnosis, and items (3), (4), and (5) were auxiliary factors for diagnosis.

GO diagnostic criteria were as follows [5]: (1) Mild GO usually involves one or more of the following symptoms: mild eyelid retraction (<2 mm), mild soft tissue damage, exophthalmos < 3 mm of the same population and sex, no diplopia or intermittent diplopia, and corneal exposure which can be improved by a lubricant, and (2) Moderate to severe GO usually has the following two or more symptoms: eyelid retraction ≥ 2 mm, moderate or severe soft tissue damage, exophthalmos ≥ the same population and sex normal value 3 mm, and persistent or intermittent diplopia.

The inclusion criteria for the experimental group were as follows: (1) initial diagnosis of GD with ophthalmopathy, (2) ophthalmopathy diagnosed as mild GO, (3) oral methimazole prescribed for GD treatment, and (4) no treatment for ophthalmopathy.

The inclusion criteria for the control group were as follows: (1) initial diagnosis of GD without ophthalmopathy and (2) oral methimazole prescribed for GD treatment.

Exclusion criteria were as follows: (1) hyperthyroidism caused by nodular goiter, thyroid cancer, high-functioning adenoma, and thyroiditis, (2) thyroidectomy, (3) primary hypothyroidism, (4) orbital inflammatory pseudotumors, orbital cellulitis, and orbital granuloma, among others, and (5) pregnancy.

The patients were divided into two groups based on changes to the eye condition: a GO-free (36 cases) group and a GO (24 cases) group. All patients were given methimazole for GD. According to EUGOGO [5], all patients in the GO group were evaluated according to the CAS. No patient scored higher than 3. Therefore, none of the patients were considered in the active disease phase. Because these patients were first diagnosed in the Endocrine Department, ocular manifestations of the disease may not have been obvious.

### 2.2. Isobaric Tags for Relative and Absolute Quantification (iTRAQ) Protein Spectrum Analysis

Peripheral venous blood was collected from patients with GO and GO-free disease and centrifuged as soon as possible to separate the serum, which was immediately stored at -80°C. High-abundance proteins were removed according to the instructions of the ProteoMiner™ kit (Bio-Rad Laboratories, USA). Protein quantification was carried out according to the BCA method, and SDS-PAGE was performed. The samples were reduced, alkylated, and enzymatically hydrolyzed. The isotope mass label iTRAQ reagent was used for labeling and high pH reverse-phase fractionation. Finally, the protein samples were analyzed using a nanoupgraded reverse-phase chromatography-TripleTOF™ 5600.

### 2.3. ELISA

Using a serum separator tube, the blood samples were allowed to clot for two hours at room temperature or overnight at 4°C before centrifugation for 20 minutes at 1000 × g. Freshly prepared serum was assayed immediately or stored in aliquots at -20°C or -80°C for later use. Experiments were performed according to the manufacturer's protocol of the FT3 ELISA kit (LEVELOP, Wuxi, China) and TRAb ELISA kit (GTX, Shanghai, China).

### 2.4. Western Blotting

Whole blood samples were centrifuged at 4°C 3000 × g for 20 minutes. The serum samples were added to serum protein extracts and centrifuged at 14,000 × g at 4°C for 10 minutes. Proteins (30 *μ*g) were separated by SDS-PAGE and transferred to polyvinylidene fluoride (PVDF) membranes (Bio-Rad Laboratories, USA). The membranes were washed for 1-2 minutes in 0.1% Tween-20 (TBST) (Solarbio, Beijing, China), blocked (5% skimmed milk powder) at 37°C for 1 hour, and incubated with a primary antibody against RhoA (1 : 5000, Abcam, UK), overnight at 4°C. After rinsing with TBST three times, the membrane was incubated with a rabbit/mouse secondary antibody at room temperature for 1 hour and exposed.

### 2.5. Biochemical Index Detection

Blood was drawn from fasting patients in the morning. Chemiluminescence immunoassays using Abbott reagents and instruments were used to measure free thyroid function. Chemiluminescence immunoassays using German Siemens reagents and instruments were employed for TPOAb and TgAb detection. Electrochemiluminescence immunoassays using Roche reagents and instruments were used to detect TRAb. The reference value range was 2.63~5.70 pmol/L for FT3, 9.01~19.05 pmol/L for FT4, 0.350~4.940 *μ*U/L for TSH, 0.00~1.75 IU/L for TRAb, 0.00~35.00 IU/mL for TPOAb, and 0.00~40.00 IU/mL for TgAb. Because the measurement range for TPOAb was 10~1000 IU/mL, a level ≥ 1000 IU/mL was considered to be 1000 IU/mL, similarly, as the range for TgAb was 20~3000 IU/mL, a level ≥ 3000 IU/mL was considered to be 3000 IU/mL.

### 2.6. Data Collection

We conducted a 7-month follow-up to record patient serum samples and clinical data from June 2018 to October 2019. Age, sex, treatment of thyroid disease, history of hyperthyroidism, and monthly levels of thyroid-related hormones (FT3, FT4, TSH, TRAb, TPOAb, and TgAb) were collected, related biochemical indexes and the time of occurrence of various eye signs were measured.

### 2.7. Statistical Analysis

Analyses were performed using SPSS version 23.0 and GraphPad Prism version 8.0 for Windows. All numerical data conforming to a normal distribution are expressed as the mean ± SD. Data not conforming to a normal distribution are presented as medians (interquartile ranges), and enumeration data are given as percentages. The Mann–Whitney *U* test was used for comparison of age. Two-tailed Fisher's exact tests were applied to compare enumeration data. Mauchly's test and the Greenhouse-Geisser correction method were utilized to determine significant differences in thyroid hormone levels of continuous data between the groups. Spearman's rank correlation coefficient was used to determine whether different hormone or antibody titer levels of the two groups correlated with the occurrence of GO. ROC curves were drawn to assess whether hormone or antibody levels predict GO pathogenesis at certain cutoff levels. The area under the ROC curve (AUC) was evaluated for diagnostic value. *P* < 0.05 was considered statistically significant.

## 3. Results

### 3.1. Demographic and Ophthalmological Data

The GO and GO-free groups were similar in age at diagnosis and sex (Supplementary Table 1).

### 3.2. Results of iTRAQ

We randomly collected venous blood from 3 GO with GD patients (experimental group) and 3 GD patients without GO (control group) with matching sex and age and performed iTRAQ protein profile analysis. As Pearson coefficients between the control group and the experimental group samples were greater than 0.86, the data were significant and repeatable.

**Figure 1:**
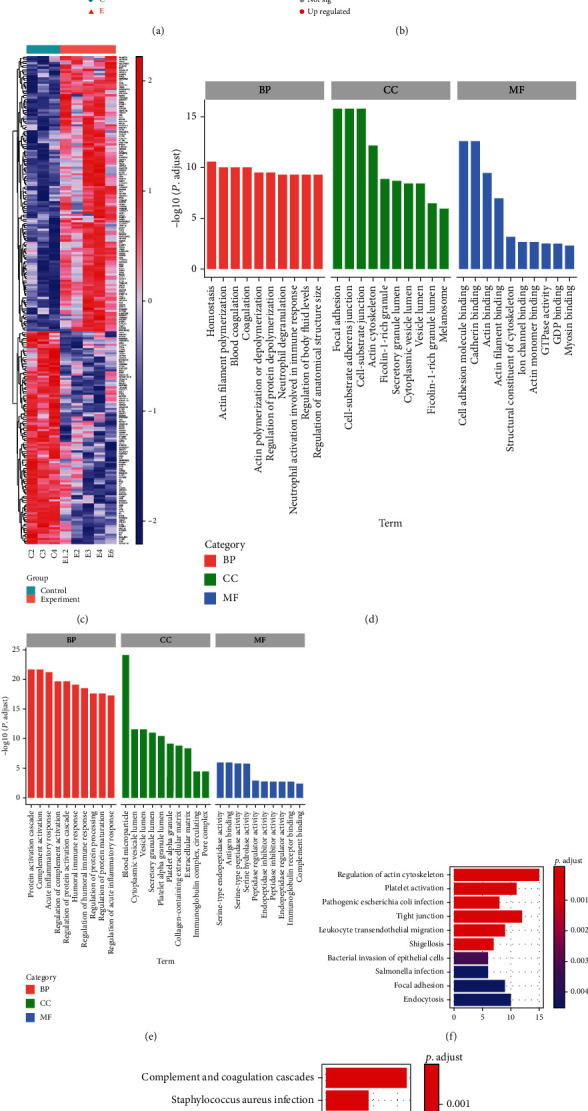
iTRAQ results. (a) Protein quantitative results were analyzed by principal component analysis. The control group and the experimental group aggregated significantly into different clusters. Blue: control group; red: experiment group. Heatmap (b) and volcano plots (c) protein expression between the control and experimental groups (fold change > 1.5 and adjusted *P* value < 0.05). Red: upregulated; blue: downregulated. (d) GO enrichment analysis was performed for upregulated proteins. The results showed that the upregulated proteins are mainly involved in actin filament polymerization, focal adhesion, and actin binding. (e) GO enrichment analysis was performed for downregulated proteins. The results showed that the downregulated proteins are mainly involved in the protein activation cascade, blood microparticles, and antigen binding. (f) KEGG pathway analysis was performed for upregulated proteins. The results showed that the upregulated proteins are mainly involved in the regulation of actin cytoskeleton, platelet activation, pathogenic Escherichia coli infection, and tight junction. (g) KEGG pathway analysis was performed for downregulated proteins. The results showed that the downregulated proteins are mainly involved in complement and coagulation cascades, Staphylococcus aureus infection, prion disease, and systemic lupus erythematosus.

In the principal component analysis of the protein quantitative results, the control group and the experimental group aggregated obviously into different clusters, indicating that protein expression in the experimental group was significantly different from that in the control group (Figure 1(a)).

We detected 1552 proteins. Compared with the control group, the experimental group showed 229 differential proteins with expression difference > 1.5. Among these, 129 proteins were upregulated and 100 downregulated in the experimental group. Among them, the most significantly upregulated proteins were YBX3, RAB3GAP2, APOC3, GCA, and CSRP2, the most significantly downregulated proteins were POLA1, SMAP, ETFB, RNMT, and MAGED4 (Figures 1(b) and 1(c)).

To better investigate the biochemical process of the occurrence and development of GO, we conducted Gene Ontology analysis of differential proteins, mainly from three aspects: cell component, molecular function, and biological process. GO analysis was performed on upregulated proteins. The functions of these targeted proteins in cell components mainly involved focal adhesions and cell substrate joints. Molecular function terms largely were cell adhesion molecule binding and actin binding. In terms of biological process, enrichment in such processes as actin polymerization and depolymerization, cell adhesion, and neutrophil-mediated immunity was observed. For downregulated differential proteins (Figure 1(e)), cell composition terms were mostly the cytoplasm and extracellular matrix. Molecular function mainly involved antigen binding and serine endopeptidase activity, and biological process terms were the protein activation cascade, complement activation, and acute inflammation.

**Figure 2:**
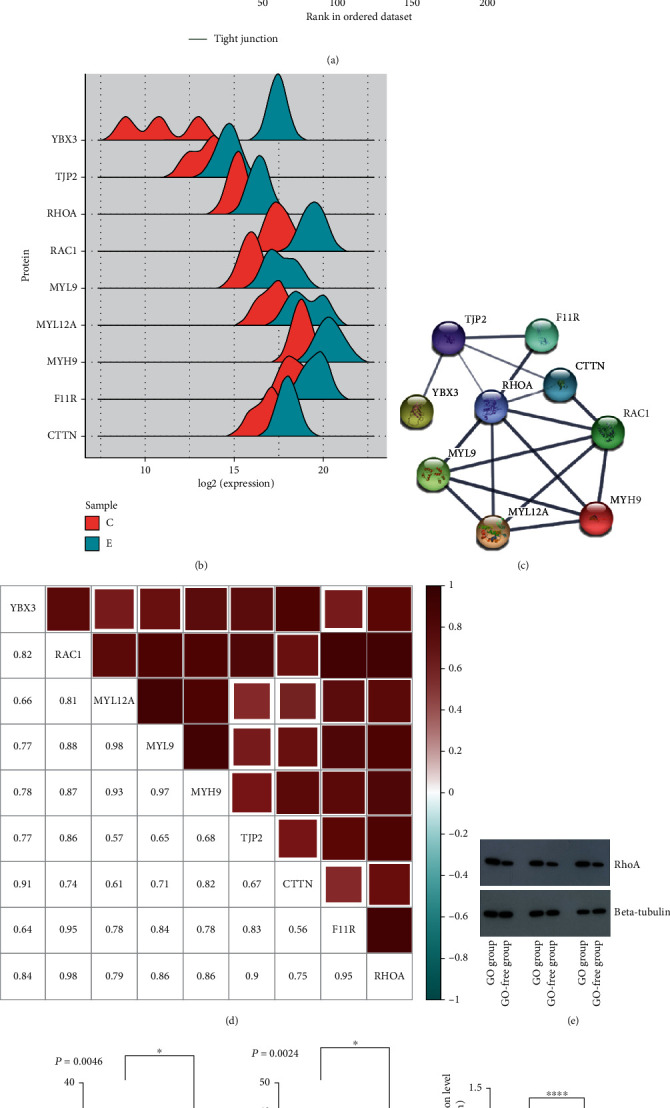
Results of bioinformatics analysis and results of ELISA and western blotting. (a) GSEA analyses of gene sets for tight junctions. (b) Ridge plot reflecting protein expression of the experimental group and the control group. Blue: experimental group; red: control group. (c) Perform protein-protein interaction analysis on differential proteins related to tight junctions, and RhoA is at the center of the interaction. (d) Pearson correlation analysis was performed on proteins involved in the tight junction pathway of GSEA. Serum samples from 3 patients with newly diagnosed GO complicated with GD (experimental group) and 3 with GD without GO (control group). These serum samples were detected by WB and ELISA. (e) Western blotting for RhoA. (f) Results of FT3 ELISA and results of TRAb ELISA. (g) The grayscale value of the RhoA band (*P* < 0.0001).

Through KEGG analysis (Figures 1(f) and 1(g)), upregulated differential proteins participate in signaling pathways such as actin cytoskeleton regulation, platelet activation, pathogenic *Escherichia coli* infection, tight junctions, and transendothelial migration of leukocytes. Downregulated proteins are mainly involved in the complement and coagulation cascade, *Staphylococcus aureus* infection, and prion diseases. We also performed gene set enrichment analysis (*P* < 0.05, FDR < 0.25) on the quantitative data for differential proteins (Figures 2(a) and 2(b)). The results showed tight junction signaling pathways to be enriched and upregulated in GO, consistent with the KEGG results. The main enriched proteins were also similar to the KEGG results, including YBX3, TJP2, RhoA, RAC1, MYL12A, MYH9, F11R, and CTTN. These proteins are all upregulated in GO.

Pearson correlation analysis revealed a high correlation of expression of proteins involved in the GSEA tight junction pathway (Figure 2(c)). High correlations, such as for RhoA, RAC1, F11R, and TJP2, suggest that these proteins may be similar in function. Furthermore, protein-protein interaction analysis of differential proteins related to tight junctions showed that RhoA is at the center of the interaction (Figure 2(d)). Next, we used western blotting to detect RhoA in serum samples from the two groups (Figure 2(e)) and calculated the grayscale value (Figure 2(g)). ELISA was applied to detect FT3 and TRAb (Figure 2(f)). The results showed that RhoA, FT3, and TRAb levels were significantly higher in the GO group than in the GO-free group.

### 3.3. Changes in RhoA, FT3, FT4, and TSH Levels

**Figure 3:**
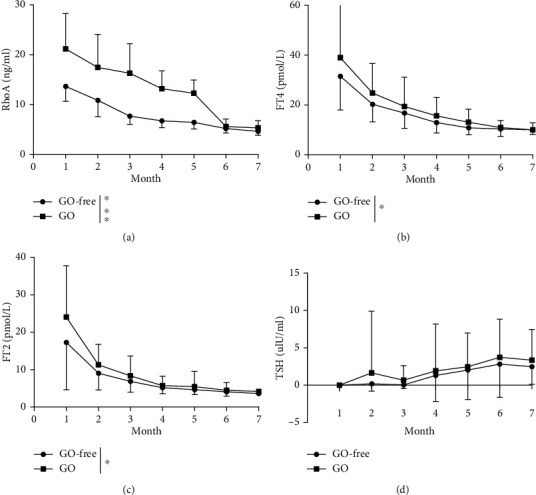
RhoA, FT3, FT4, and TSH levels in patients in the GO-free and GO groups over a 7-month follow-up period. (a–c) After eliminating the interaction between hormones and time by the Greenhouse-Geisser correction method, monthly RhoA, FT4, and FT3 levels in the GO group were higher than those in the GO-free group. (d) After eliminating the interaction of hormones and time by the Greenhouse-Geisser correction method, no significant differences in TSH levels were observed between the GO-free and GO groups.

In the GO-free group, 24 patients (66.7%) developed hypothyroidism, with reversion to normal thyroid function levels occurring in 12 (33.3%). In the GO group, 20 patients (83.3%) had hypothyroidism, and 4 experienced reversions to normal thyroid function levels (16.7%). Overall, changes in the thyroid hormone levels of the two groups of patients were recorded for 7 consecutive months (Figures 3(b)–3(d)), and serum RhoA levels were assessed by ELISA (Figure 3(a)). We performed repeated-measurement data analysis of variance for FT3, FT4, TSH, and RhoA at different time points. Mauchly's test of sphericity was performed on factors in each group, however, the data did not satisfy the sphere hypothesis (*P* < 0.05, Supplementary Table 2). Hence, the values were adjusted using the Greenhouse-Geisser correction method (Supplementary Tables 2 and 3). A correlation between repeated measurements of FT3, FT4, TSH, and RhoA data was detected. After eliminating the interaction of hormones and time, FT3, FT4, and RhoA levels in the GO-free and GO groups were statistically significant, though there were no significant differences in TSH. Nevertheless, monthly FT3, FT4, and RhoA levels were higher in the GO group than in the GO-free group.

### 3.4. Changes in Basal Levels, Endpoint Values, and Differences in Thyroid-Related Hormone and RhoA Levels

For the two groups, we calculated differences in hormone and RhoA levels between baseline and the follow-up endpoint to evaluate fluctuations (Table 1). Numerical data in accordance with a normal distribution are expressed as the mean ± SD, and the parameter *t*-test was used. Data that did not conform to a normal distribution are presented as the median (range of quartiles), and the Mann–Whitney *U* test was used. The difference between basal levels and endpoint values was statistically significant for RhoA and FT3 levels but not for FT4 or TSH.

### 3.5. Changes in Thyroid Antibody Titer at the First Diagnosis of GD

**Figure 4:**
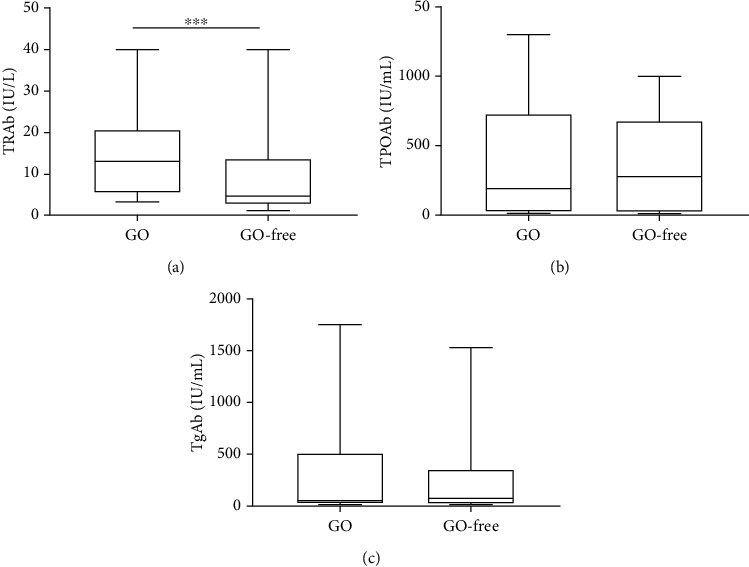
Differences in thyroid antibody titer at first diagnosis of GD. (a) The difference in TRAb titer levels was statistically significant (*P* = 0.004). (b, c) TPOAb (*P* = 0.972) and TgAb (*P* = 0.929) titer levels were not statistically significant. Median (line), interquartile range (box), and total range (whiskers) for TRAb, TPOAb, and TgAb titers between the GO-free group and the GO group when the two groups of patients were diagnosed with GD.

Because TRAb levels change slightly during short-term treatment and TPOAb and TgAb are not affected by antithyroid drugs, we compared antibody titers of the two groups of patients at first diagnosis of GD using the Mann–Whitney *U* test. The difference in TRAb titer (*P* = 0.004) was statistically significant (Figure 4(a)), whereas that for TPOAb and TgAb titers (*P* = 0.973, *P* = 0.929) was not (Figures 4(b) and 4(c)).

### 3.6. Correlation Analysis of Thyroid Hormone and Related Antibodies with GO

Thyroid hormone levels, antibody titers, and RhoA levels were analyzed using Spearman rank correlation analysis, revealing that basal RhoA levels correlated positively with the incidence of GO. In addition, the higher the basal RhoA level was, the more likely the patient was to develop GO. Moreover, the difference between the RhoA endpoint value and basal level correlated negatively with the incidence of GO. As the absolute value was greater when there was a smaller difference between the RhoA endpoint value and the basal level, such patients were likely to develop GO. In addition, basal FT3 levels correlated positively with GO incidence, where the greater the basal FT3 level was, the more prone the patient was to developing GO. The difference between the FT3 endpoint value and basal level correlated negatively, with a smaller difference between the FT3 endpoint value and basal level indicating a greater absolute value and likely GO development. TRAb titers at first diagnosis of GD also correlated positively with the incidence of GO, and patients with a large TRAb value were prone to develop GO. Conversely, FT4, TSH, TPOAb, and TgAb levels did not correlate with GO onset (Table 2).

### 3.7. Diagnostic Significance of RhoA, FT3, FT4, TSH, and Related Antibodies in Onset of GO

**Figure 5:**
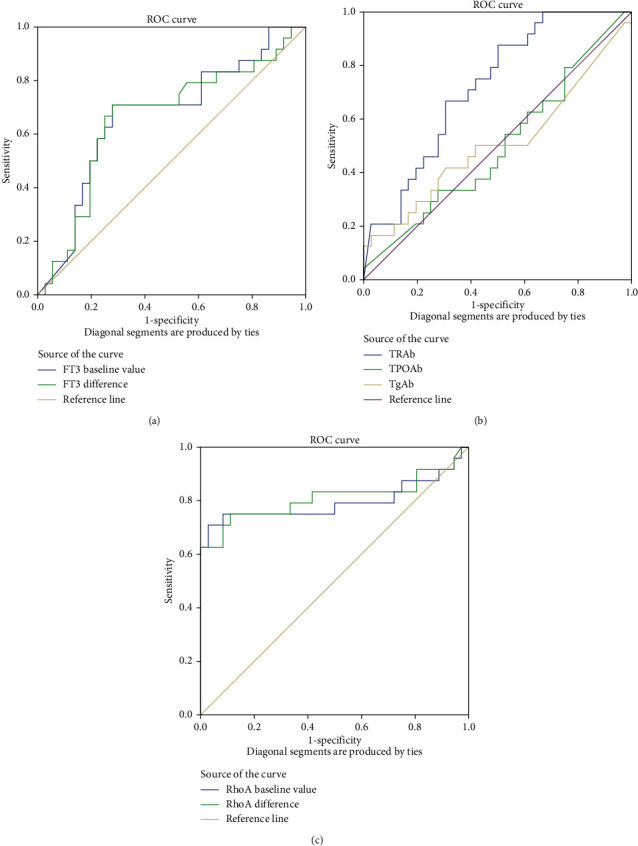
Receiver operating characteristic (ROC) curves were generated to evaluate the diagnostic significance of RhoA, FT3, FT4, TSH, TPOAb, and TgAb for onset of GO. (a) The area under the ROC curve (AUC) refers to the area formed by the ROC curve and the *x*-axis, (1, 0) -(1, 1). The basal FT3 level and difference between the basal FT3 level and endpoint value had diagnostic value. (b) TRAb titer also had diagnostic value and was superior to FT3. FT4, TSH, TPOAb, and TgAb titers had no diagnostic value. (c) The basal RhoA level and difference between the basal RhoA level and endpoint value had diagnostic value.

We used specificity as the horizontal axis and sensitivity as the vertical axis to generate a ROC curve to evaluate the diagnostic significance of thyroid hormones, RhoA, and related antibodies. The results demonstrated that basal FT3 levels, basal RhoA levels, and the difference between basal FT3 levels and FT3 endpoint values and the difference between basal RhoA and RhoA endpoint values had diagnostic values (the difference indicates the absolute value) (Figure 5(a)). When patients were diagnosed with GD, TRAb titers also had diagnostic values and were superior to FT3. However, FT4, TSH, TPOAb, and TgAb levels exhibited no diagnostic value (Figure 5(b) and Table 2).

## 4. Discussion

GO is an autoimmune disease and a common orbital disease that affects vision eye movement and appearance. GO is seriously detrimental to quality of life. The incidence rate of GO is approximately 16 females and 3 males per 100,000 people [11]. Most patients with GD are overlooked due to the mild ocular symptoms, and treatment tends to focus on GD, delaying the diagnosis and treatment of GO. Hence, we aimed to identify biomarkers for early GO diagnosis through iTRAQ analysis. We investigated GD patients with new-onset GO to determine predisposing factors influencing the occurrence of GO. We used iTRAQ and WB and identified that expression of RhoA was significantly increased in GO, as verified in 60 patients and found that expression of RhoA was significantly higher in the GD with GO group than in the GD group at the initial stage (basal level = 25.09, *P* < 0.001).

Ras homolog family member A (RhoA) is a low-molecular-weight G protein, and serine/threonine kinase Rho-associated kinase (ROCK) is one of the main downstream effectors of RhoA [15]. Studies have shown that RhoA plays important roles in cell adhesion, migration, and transformation, which is consistent with the results of our KEGG analysis. For example, Wei et al. [16] studied an in vitro model of GO and found that RhoA is involved in these processes. The RhoA/ROCK signaling pathway is also involved in the differentiation of myofibroblasts induced by TGF-*β*. And in the pathogenesis of GO, orbital fibroblasts are the central target of the immune response and are involved in the process of inflammation and remodeling of orbital connective tissue and extraocular muscles. GO is caused by an autoimmune reaction of shared antigens between the thyroid and the orbit, mainly involving the connective tissue behind the bulb and extraocular muscles. Based on immunohistochemical experiments of orbital tissues of patients with GO [17, 18], Weetman et al. [19] reported infiltration of a large number of lymphocytes, neutrophils, serum cells, and mucopolysaccharide deposits, mainly in fat cells and intermuscular spaces, and that the lymphocytes were T cells. It has also been found that the Th1/Th2 balance participates in the progression of GO, with a shift toward Th1 dominance in the later stage [20]. RhoA is a ubiquitously expressed cytoplasmic protein belonging to the small GTPase family and plays a key regulatory role in innate and adaptive immunity. For example, many scientists have shown that Th0 cells differentiate into one of four main subgroups (Th1, Th2, Th17, and Treg) to perform functions when the initial T lymphocytes entering the periphery are activated by the dual signals of antigen-presenting cells [21–23]. Tregs play the most important role in regulating self-reactive T cells and in preventing an excessive immune reaction, which is harmful to the host [21]. RhoA is one of the core proteins for T cell migration [23], and studies on T cell-specific knockout RhoA mice show that RhoA is vital for T cell proliferation, activation, and migration. Therefore, we speculate that RhoA affects the balance of Th1/Th2 cells by interrupting Th0 cell differentiation, which promotes GO pathogenesis.

With the gradual development of GO, severe exophthalmos can lead to incomplete eyelid closure, dry exfoliation of the corneal epithelium, exposure keratitis, corneal ulcers, and even corneal perforation. Hypertrophic extraocular muscles oppress the optic nerve, which can lead to optic neuropathy and even vision loss. ROCK inhibitors have been shown to promote corneal cell proliferation and repair [24, 25]. Okumura et al. [26] found that apoptosis can activate phosphorylation within the Rho/ROCK signaling pathway in corneal endothelial cells, and the loss of cell adhesion can induce apoptosis. ROCK inhibitors can counteract the loss of cell adhesion by activating the focal adhesion complex. Inhibition of the Rho/ROCK pathway can also promote axonal regeneration and functional repair of the central nervous system. Yu et al. [27] established a rabbit model of optic nerve injury and administered the Rho/ROCK inhibitor fasudil. The state of retinal ganglion cells was better in the model group than in the saline group or the dexamethasone group, suggesting that inhibition of the Rho/ROCK signaling pathway can promote optic nerve repair to some extent. Furthermore, we observed increases in TRAb in 60 patients with GD and GO compared with GD patients. GD is characterized by the presence of antibodies against TSHR [28–30]. TSH-receptor antibodies (TRAbs) are divided into thyroid-stimulating (TSAbs), thyroid-blocking (TBAbs), and neutral (neutral Abs) antibodies. Studies have demonstrated a close clinical and temporal relationship between GD and GO, which suggests a common pathogenic antigen in the thyroid and orbit [4, 5, 31]. As TRAb is an autoantibody that causes GD [32], TRAb levels may have a certain clinical value in determining the relationship between GD and GO [33]. In our study, the average TRAb titer in the GO-free group at first diagnosis of GD was 4.58 (IU/L), whereas the titer in patients in the GO group was 13.07 (IU/L), indicating diagnostic significance for GO (Figures 2(c) and 3(b)). Our findings are consistent with those of several published studies [34, 35]. It has also been shown that levels of TSAb and TBII may be related to the incidence and severity of GO, with more significance for the former [34, 36]. At present, it is believed that a lack of CD28 on the surface of CD4+ and CD8+ T cell subsets in the peripheral blood is the reason that patients are prone to immune-mediated diseases [37, 38]. The amount of CD28 in the serum of patients with GD is higher than that of normal subjects, and levels of endogenous IFN-*γ* and IL-6 cytokines in patients with GD with GO are higher than those in GO-free patients, with a positive correlation with TRAb [37–41].

TSH is the most sensitive indicator for thyroid function. As T3 and T4 levels are usually affected by binding proteins and albumin, FT3 and FT4 can well reflect thyroid function. During the 7-month follow-up, we found that basal FT3 levels and the difference between basal levels and endpoint values had diagnostic values for GO, based on Mann–Whitney *U* test results (AUC = 0.669, *P* = 0.028; AUC = 0.660, *P* = 0.037). Nonetheless, there were no statistically significant differences between the endpoints of FT4 and TSH and their basal levels. Thus, a true representation of thyroid function should be evaluated using serum FT4 levels instead of serum FT3 levels. However, in our study, FT3 levels in the serum of GO patients showed a positive result that was significantly different from that for the serum of GO-free patients, which may be because FT3 is more sensitive than FT4 during the early stages. We collected patient serum during the early stages of GD, and FT3 might be more easily overactivated than FT4. Hence, patients with GD with significantly elevated FT3 during the early stages were more likely to develop GO, therefore, such an increase in FT3 was easy to detect.

Based on our study, we speculate that RhoA is a potential biomarker for GO and may play an important role in the differentiation and migration of T cells and promote the pathogenesis of GO. We also found that GD patients with a significant increase in TRAb and FT3 in the early stage were more likely to develop GO. TRAb is an autoantibody that is highly expressed in the adipose tissue of GO patients and correlates positively with the secretion of endogenous INF-*γ* and IL-6 cytokines [42]. Therefore, we speculate that RhoA and TRAb promote the development of GO at the immune level, but whether there is a direct relationship between them requires more detailed evidence

## 5. Limitation

Relatively few patients met the inclusion criteria, and some patients were excluded because of loss to follow-up, poor compliance, and the use of other oral drugs. These factors may have affected the results of the experiment. Therefore, the study was limited by the inadequate sample size, which we will aim to expand by continuing to collect relevant medical records. At the same time, the conclusions we reached based on the current sample size are consistent with those drawn by others, indicating that the results of this experiment are credible despite the limitations.

## 6. Conclusion

RhoA is a potential biomarker in the mild stage of GO. If elevation of RhoA in the serum of GD patients is accompanied by elevation of TRAb or FT3, GO is highly likely to occur, even in the absence of obvious ocular symptoms. Our study provides a new biomarker for early diagnosis of GO, and we hope that it will allow patients with GO to be diagnosed and treated in a timelier manner in the future.

## Figures and Tables

**Table 1:** Comparison of FT3\FT4\TSH endpoint values and baseline values between the two groups of patients.

Parameters	GO-free	GO	*P* value
Basal RhoA level	13.63 ± 2.99	25.09 (14.77, 26.11)	**<0.001**
Endpoint RhoA level	5.16 ± 0.86	5.59 ± 1.50	0.1643
RhoA difference	−8.47 ± −15.59	-18.03 (-20.69, -10.95)	**<0.001**
Basal FT3 level	13.00 (8.81, 17.17)	21.82 (12.35, 36.38)	**0.027**
Endpoint of FT3	3.89 (3.36, 4.71)	3.97 (3.60, 4.92)	0.407
FT3 difference	-9.35 (-13.28, -5.17)	-17.45 (-31.18, -8.93)	**0.036**
Basal FT4 levels	28.08 (23.24, 33.60)	30.72 (25.38, 43.33)	0.215
Endpoint of FT4	10.38 (8.87, 11.24)	10.29 (8.27, 12.91)	0.979
FT4 difference	-17.83 (-24.84, -12.58)	-21.52 (-33.59, -15.25)	0.130
Basal TSH levels	0.004 (0.004, 0.004)	0.004 (0.004, 0.004)	0.960
Endpoint of TSH	0.442 (0.142, 3.889)	2.425 (0.048, 5.209)	0.581
TSH difference	0.440 (0.100, 3.878)	2.425 (0.045, 5.203)	0.546

RhoA difference: difference between the RhoA endpoint value and the basal level. FT3 difference: difference between the FT3 endpoint value and the basal level. FT4 difference: difference between the FT4 endpoint value and basal level. TSH difference: difference between the TSH endpoint value and basal level. Statistically significant results are in bold.

**Table 2:** Correlation coefficients and diagnostic significance of thyroid hormone and related antibodies with GO incidence for the two groups.

Parameters	*r*	*P* value	Area under the curve (AUC)	*P* value	Confidence interval of 0.95	
					Lower bound	Upper bound
Basal RhoA levels	0.501	**0.001**	0.795	**0.001**	0.652	0.938
Endpoint RhoA levels	0.197	0.130	0.616	0.129	0.459	0.774
RhoA difference	-0.527	**0.001**	0.811	**0.001**	0.676	0.945
Basal FT3 levels	0.287	**0.026**	0.669	**0.028**	0.526	0.812
Endpoint value of FT3	0.109	0.407	0.564	0.402	0.417	0.711
FT3 difference	-0.272	**0.035**	0.660	**0.037**	0.513	0.807
Basal FT4 levels	0.163	0.213	0.596	0.210	0.445	0.747
Endpoint value of FT4	0.004	0.976	0.502	0.976	0.346	0.658
FT4 difference	-0.198	0.129	0.617	0.128	0.469	0.764
Basal TSH levels	0.019	0.886	0.508	0.922	0.358	0.657
Endpoint value of TSH	0.073	0.581	0.543	0.577	0.388	0.697
TSH difference	0.080	0.545	0.547	0.541	0.393	0.700
TRAb	0.372	**0.003**	0.719	**0.004**	0.592	0.846
TPOAb	0.005	0.970	0.497	0.970	0.346	0.648
TgAb	0.012	0.926	0.507	0.928	0.349	0.665

Statistically significant results are in bold.

## Data Availability

The data used to support the findings of this study are available from the corresponding authors upon request.

## Supplementary Materials

Supplementary Materials Supplementary Table 1: demographic and ophthalmological data of the study participants, i.e., GO-free and GO groups. Supplementary Table 2: Mauchly's sphericity test of thyroid hormone levels in the two groups. Supplementary Table 3: analysis of variance of repeated-measurement data for the two groups.

## References

[1] Bartalena L. (2013). Diagnosis and management of Graves disease: a global overview. *Nature Reviews Endocrinology*.

[2] Ross D. S., Burch H. B., Cooper D. S. (2016). 2016 American Thyroid Association guidelines for diagnosis and management of hyperthyroidism and other causes of thyrotoxicosis. *Thyroid*.

[3] Wang L., Wang F.-S., Gershwin M. E. (2015). Human autoimmune diseases: a comprehensive update. *Journal of Internal Medicine*.

[4] Şahlı E., Gündüz K. (2017). Thyroid-associated ophthalmopathy. *Türk Oftalmoloji Dergisi*.

[5] Bartalena L., Baldeschi L., Boboridis K. (2016). The 2016 European Thyroid Association/European Group on Graves' orbitopathy guidelines for the management of Graves' orbitopathy. *European Thyroid Journal*.

[6] Piantanida E., Tanda M. L., Lai A., Sassi L., Bartalena L. (2013). Prevalence and natural history of Graves' orbitopathy in the XXI century. *Journal of Endocrinological Investigation*.

[7] Leo M., Menconi F., Rocchi R. (2015). Role of the underlying thyroid disease on the phenotype of Graves' orbitopathy in a tertiary referral center. *Thyroid*.

[8] Bartley G. B., Gorman C. A. (1995). Diagnostic criteria for Graves' ophthalmopathy. *American Journal of Ophthalmology*.

[9] Milbratz G. H., Garcia D. M., Guimarães F. C., Cruz A. A. V. (2012). Multiple radial midpupil lid distances: a simple method for lid contour analysis. *Ophthalmology*.

[10] Bahn R. S., Heufelder A. E. (1993). Pathogenesis of Graves' ophthalmopathy. *The New England Journal of Medicine*.

[11] Bahn R. S. (2010). Graves' ophthalmopathy. *New England Journal of Medicine*.

[12] Sun S., Poon R. T. P., Lee N. P. (2010). Proteomics of hepatocellular carcinoma: serum vimentin as a surrogate marker for small tumors (≤2 cm). *Journal of Proteome Research*.

[13] Li N., Long Y., Fan X. (2009). Proteomic analysis of differentially expressed proteins in hepatitis B virus-related hepatocellular carcinoma tissues. *Journal of Experimental & Clinical Cancer Research*.

[14] Ignjatovic V., Geyer P. E., Palaniappan K. K. (2019). Mass spectrometry-based plasma proteomics: considerations from sample collection to achieving translational data. *Journal of Proteome Research*.

[15] Tan H.-B., Zhong Y.-S., Cheng Y., Shen X. (2011). Rho/ROCK pathway and neural regeneration: a potential therapeutic target for central nervous system and optic nerve damage. *International Journal of Ophthalmology*.

[16] Wei Y.-H., Liao S.-L., Wang S.-H., Wang C.-C., Yang C.-H. (2021). Simvastatin and ROCK inhibitor Y-27632 inhibit myofibroblast differentiation of Graves’ ophthalmopathy-derived orbital fibroblasts via RhoA-mediated ERK and p38 signaling pathways. *Frontiers in Endocrinology*.

[17] Smith T. J., Janssen J. A. M. J. (2019). Insulin-like growth factor-I receptor and thyroid-associated ophthalmopathy. *Endocrine Reviews*.

[18] Smith T. J. (2019). Challenges in orphan drug development: identification of effective therapy for thyroid-associated ophthalmopathy. *Annual Review of Pharmacology and Toxicology*.

[19] Weetman A. P., Cohen S., Gatter K. C., Pells P., Shine B. (1989). Immunohistochemical analysis of the retrobulbar tissues in Graves' ophthalmopathy. *Clinical and Experimental Immunology*.

[20] Xia N., Zhou S., Liang Y. (2006). CD4+ T cells and the Th1/Th2 imbalance are implicated in the pathogenesis of Graves' ophthalmopathy. *International Journal of Molecular Medicine*.

[21] Bros M., Haas K., Moll L., Grabbe S. (2019). RhoA as a key regulator of innate and adaptive immunity. *Cells*.

[22] Gagliani N., Huber S. (2017). *Basic Aspects of T Helper Cell Differentiation*.

[23] Zhu J., Paul W. E. (2010). Peripheral CD4+ T-cell differentiation regulated by networks of cytokines and transcription factors. *Immunological Reviews*.

[24] Ljubimov A. V., Saghizadeh M. (2015). Progress in corneal wound healing. *Progress in Retinal and Eye Research*.

[25] Syed Z. A., Rapuano C. J. (2021). Rho kinase (ROCK) inhibitors in the management of corneal endothelial disease. *Current Opinion in Ophthalmology*.

[26] Okumura N., Fujii K., Kagami T. (2016). Activation of the rho/rho kinase signaling pathway is involved in cell death of corneal endothelium. *Investigative Ophthalmology & Visual Science*.

[27] Yu J., Lin L., Luan X., Jing X., Maierab (2015). Impacts of rho kinase inhibitor fasudil on rho/ROCK signaling pathway in rabbits with optic nerve injury. *International Journal of Clinical and Experimental Pathology*.

[28] Träisk F., Tallstedt L., Abraham-Nordling M. (2009). Thyroid-associated ophthalmopathy after treatment for Graves’ hyperthyroidism with antithyroid drugs or iodine-131. *The Journal of Clinical Endocrinology & Metabolism*.

[29] Chen D. Y., Schneider P. F., Zhang X. S., Luo X. Y., He Z. M., Chen T. H. (2014). Changes in Graves' ophthalmopathy after radioiodine and anti-thyroid drug treatment of Graves' disease from 2 prospective, randomized, open-label, blinded end point studies. *Experimental and Clinical Endocrinology & Diabetes*.

[30] Fröhlich E., Wahl R. (2017). Thyroid autoimmunity: role of anti-thyroid antibodies in thyroid and extra-thyroidal diseases. *Frontiers in Immunology*.

[31] Bartalena L., Baldeschi L., Dickinson A. (2008). Consensus statement of the European Group On Graves' Orbitopathy (EUGOGO) on management of GO. *European Journal of Endocrinology*.

[32] Chen X., Huang F., Qi Y. (2018). Serum and thyroid tissue level of Let-7b and their correlation with TRAb in Graves’ disease. *Journal of Translational Medicine*.

[33] Jang S. Y., Shin D. Y., Lee E. J., Choi Y. J., Lee S. Y., Yoon J. S. (2013). Correlation between TSH receptor antibody assays and clinical manifestations of Graves' orbitopathy. *Yonsei Medical Journal*.

[34] Eckstein A. K., Plicht M., Lax H. (2006). Thyrotropin receptor autoantibodies are independent risk factors for Graves’ ophthalmopathy and help to predict severity and outcome of the disease. *The Journal of Clinical Endocrinology & Metabolism*.

[35] Williams D. E., Le S. N., Godlewska M., Hoke D. E., Buckle A. M. (2018). Thyroid peroxidase as an autoantigen in Hashimoto's disease: structure, function, and antigenicity. *Hormone and Metabolic Research*.

[36] Subekti I., Boedisantoso A., Moeloek N. D., Waspadji S., Mansyur M. (2012). Association of TSH receptor antibody, thyroid stimulating antibody, and thyroid blocking antibody with clinical activity score and degree of severity of Graves ophthalmopathy. *Acta Medica Indonesiana*.

[37] Sharpe A. H., Freeman G. J. (2002). The B7-CD28 superfamily. *Nature Reviews Immunology*.

[38] Salomon B., Bluestone J. A. (2001). Complexities of CD28/B7: CTLA-4 costimulatory pathways in autoimmunity and transplantation. *Annual Review of Immunology*.

[39] Sun Z., Yi L., Tao H. (2014). Clinical immunology enhancement of soluble CD28 levels in the serum of Graves' disease. *Central European Journal of Immunology*.

[40] Kumar S., Schiefer R., Coenen M. J., Bahn R. S. (2010). A stimulatory thyrotropin receptor antibody (M22) and thyrotropin increase interleukin-6 expression and secretion in Graves' orbital preadipocyte fibroblasts. *Thyroid*.

[41] Eliana F., Suwondo P., Asmarinah A. (2017). The role of cytotoxic T-lymphocyte-associated protein 4 (CTLA-4) gene, thyroid stimulating hormone receptor (TSHR) gene and regulatory T-cells as risk factors for relapse in patients with Graves disease. *Acta Medica Indonesiana*.

[42] Khoo T. K., Bahn R. S. (2007). Pathogenesis of Graves' ophthalmopathy: the role of autoantibodies. *Thyroid*.

