# Supplementary material for: RhoA with Associated TRAb or FT3 in the Diagnosis and Prediction of Graves' Ophthalmopathy
Source: Dis Markers. 2022 Jul 29;2022:8323946. doi: 10.1155/2022/8323946 (PMC9355757; doi:10.1155/2022/8323946)
Supplement: Supplementary Materials — Supplementary Table 1: demographic and ophthalmological data of the study participants, i.e., GO-free and GO groups. Supplementary Table 2: Mauchly's sphericity test of thyroid hormone levels in the two groups. Supplementary Table 3: analysis of variance of repeated-measurement data for the two groups. [file 8323946.f1.docx]

Supplementary Table 1 Demographic and ophthalmological data of the study participants, i.e., GO-free and GO group

| Parameters | GO group  （n=24） | GO-free group(n=36) | P value |
| --- | --- | --- | --- |
| Age at diagnosis（year） | 38.04±14.16 | 41.25±13.10 | 0.312 |
| Females, n (%) | 20(83.3) | 31(86.1) | 1.000 |

P value<0.05 was considered statistically significant.

Supplementary Table 2 Mauchly's sphericity test of thyroid hormone levels in the two groups

| Within-subject effect | Mauchly's W | Approximate chi-square | df | P value |
| --- | --- | --- | --- | --- |
| FT3 | 0.001 | 560.730 | 20 | 0.001 |
| FT4 | 0.001 | 393.630 | 20 | 0.001 |
| TSH  RhoA | 0.031  0.013 | 193.006  243.038 | 20  20 | 0.001  0.001 |

Supplementary Table 3 Analysis of variance of repeated measurement data for the two groups

| Parameters | F | P-value |
| --- | --- | --- |
| FT3 | 6.275 | **0.015** |
| FT4 | 4.538 | **0.037** |
| TSH  RhoA | 2.608  80.129 | 0.112  **0.001** |

Statistically significant results in **bold**.
